# The role of pro-domains in human growth factors and cytokines

**DOI:** 10.1042/BST20200663

**Published:** 2021-09-08

**Authors:** Matthew Ratcliff, Richard Xu Zhou, Lutz Jermutus, Marko Hyvönen

**Affiliations:** 1 Department of Biochemistry, University of Cambridge, 80 Tennis Court Road, Cambridge CB2 1GA, U.K.; 2 Research & Early Development, Cardiovascular, Metabolism and Renal Diseases (CVRM), BioPharmaceuticals R&D, AstraZeneca, Cambridge, U.K.

**Keywords:** cytokine, growth factor, precursor, pro-domain, processing

## Abstract

Many growth factors and cytokines are produced as larger precursors, containing pro-domains, that require proteolytic processing to release the bioactive ligand. These pro-domains can be significantly larger than the mature domains and can play an active role in the regulation of the ligands. Mining the UniProt database, we identified almost one hundred human growth factors and cytokines with pro-domains. These are spread across several unrelated protein families and vary in both their size and composition. The precise role of each pro-domain varies significantly between the protein families. Typically they are critical for controlling bioactivity and protein localisation, and they facilitate diverse mechanisms of activation. Significant gaps in our understanding remain for pro-domain function — particularly their fate once the bioactive ligand has been released. Here we provide an overview of pro-domain roles in human growth factors and cytokines, their processing, regulation and activation, localisation as well as therapeutic potential.

## Introduction

Regulation of biological processes is critical for the correct functioning of an organism. Proteins are often at the heart of this regulation, and mechanisms used for controlling their activities are many-fold. One such mechanism by which the activity of a protein can be controlled is via the inclusion of a pro-domain or pro-peptide (see Table 1 for nomenclature we use). This is by no means an unusual mode of regulation as almost 1 in 25 human proteins are known to contain a pro-peptide or pro-domain (756 out of 20 394 of annotated human proteins in UniProt contain ‘pro-domain’ and ‘pro-peptide’ keywords). These proteins are initially produced as inactive precursor polypeptides, which are subsequently rendered active in a spatially and temporally appropriate way through proteolytic cleavage between the pro-domain and the mature growth factor (GF). Such activation of precursor proteins through proteolytic processing is well characterised for proteases, which are often produced as inactive precursors, also called zymogens, with pro-domain often serving as both a folding chaperone and an inhibitor [1], but less appreciated in the context of growth factors.

**Table 1:** Jargon buster

Term	Definition
Growth factor (GF)	Bioactive protein that can activate signalling through receptor binding.
Mature domain	GF from which pro-domain has been removed and that can bind to receptor, activating signalling.
Pro-domain	A removable domain distinct from the mature GF, dispensable for bioactivity but possibly playing some functional role, most typically, inhibition while still tethered to the GF.
Pro-peptide	A pro-domain that is not predicted to form a globular, folded structure on its own.
Precursor	Uncleaved form of the growth factor with pro- and mature domains still as a single polypeptide.
Pro-form or pro-complex	Full-length protein including both pro- and mature domains, e.g. pro-activin A, cleaved by a protease. The mature and pro-domains remain non-covalently attached.

Intercellular signalling is a process by which different parts of a multicellular organism communicate with each other. This is typically achieved with the aid of secreted proteins, growth factors, cytokines and hormones, which are released by the cell that synthesises them. They then diffuse to the target cell that presents the appropriate receptor, triggering a signalling cascade. Pro-domain function is relatively well studied in the Transforming Growth Factor β (TGF-β) family of growth factors, however numerous other proteins contain pro-domains of both known and unknown function. In this review, we take a broader look at all families of human growth factors and cytokines and see how prevalent pro-domains are, what they look like in sequence and how they contribute to the biological functions of these proteins.

## Survey of growth factor pro-domains

We searched the UniProt protein database (www.uniprot.org, version 2020_06) with keywords ‘pro-domain’ and ‘pro-peptide’ to find secreted human signalling proteins (growth factors, cytokines and hormones) that are synthesised as larger precursors. As annotation can be variable and nomenclature is not always consistent, we then expanded the search manually looking for other members of already identified protein families and other similar proteins we expected to be in the list. We also limited the search to folded proteins, leaving out peptide hormones which are not expected to have defined three-dimensional structure in isolation. We also excluded possible splice variants from this analysis.

**Figure 1.**
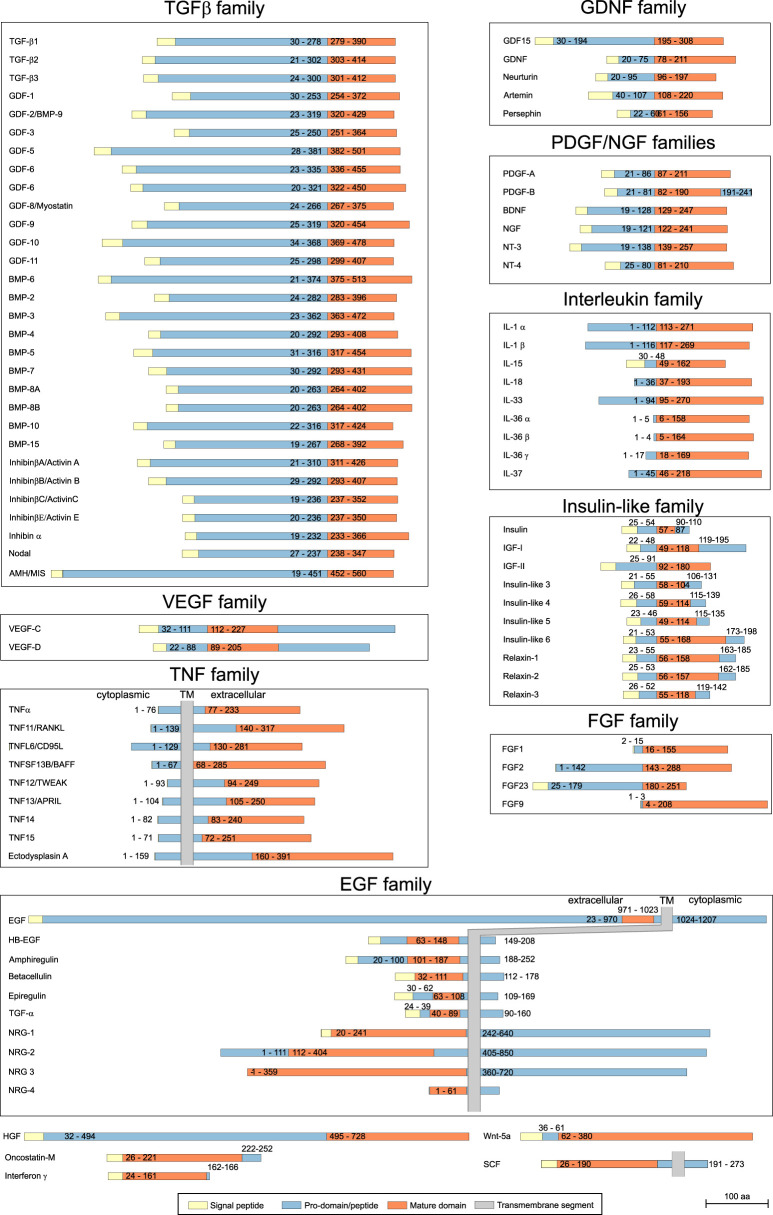
Primary structure diversity of precursor forms in human growth factors and cytokines. Representative pro-domains from different families of growth factors and cytokines are shown as schematic diagrams, in scale, based on their sequence annotation in the UniProt sequence database and our manual curation. Colouring of different parts is described at the bottom of the figure, the numbers refer to start and end residues of the different domains in the canonical isoforms in Uniprot database and the scale bar on bottom right displays the length of 100 residues in the diagrams.

The results of this data gathering are shown schematically in Figure 1. The most identifiable feature is that pro-domains vary greatly in size. In many cases the pro-domains are as big or even much bigger than the mature ligand, with epidermal growth factor (EGF) being the most extreme in this respect; EGF is synthesised as a protein of more than 1200 residues, but the bioactive mature GF is only 52 residues. Pro-domains or -peptides are more often found in the N-terminal part of the protein, but they can also be C-terminal, or the mature domain is flanked by removable parts at both ends. The insulin family is unique in containing a so-called C-peptide in the middle of the precursor, resulting in disulfide-linked dimers containing N- and C-terminal parts of the precursor. Precursors of the EGF and tumour necrosis factor α (TNF-α) family proteins and stem cell factor (SCF) contain well-predicted transmembrane segments and can consequently exist both as soluble ligands as well as bioactive membrane-bound proteins [2–4]. Some of the interleukins lack a signal peptide, which would be expected in secreted proteins, as they are initially produced intracellularly until processed by proteases and then secreted [5]. Hepatocyte growth factor (HGF) is in a class of its own: it is made as an inactive pro-HGF and activated proteolytically in the extracellular space, but the two parts remain attached by a disulfide bond with both parts needed for bioactivity [6]. This diversity alone provokes questions about the function and role of the precursor forms and pro-domains in these proteins, yet surprisingly little is known compared to the function of the mature GFs.

While we have not done comprehensive analysis of sequence conservation in the pro-domains, it is clear that these are less conserved across the species than the mature domains are. Also, while the domain structure, as shown in Figure 1, shows apparent conservation within a protein family, at the sequence level the conservation of pro-domains can be low or even non-existent. This is perhaps not surprising as the mature GFs are often signalling through shared receptors while the pro-domains and -peptides have been able to differentiate to play more specialised roles in these proteins.

In the following, we will discuss some of the growth factor families which are produced in a precursor form and for which information about processing and the role of pro-domains are available.

## The TGF-β family

The TGF-β family of proteins contain large pro-domains, typically 2–3 times larger than the mature ligands, which are known to play a key role in the activity of the mature growth factors [7]. It is perhaps the best studied group in this respect and one where most structural information is available. The genes for these growth factors encode for a 210–432 residues pro-domain and 100–120 residues mature GF ligand. Typically, the precursor is processed by furin-like proteases in the Golgi complex, and cells secrete a processed pro-form.

**Figure 2.**
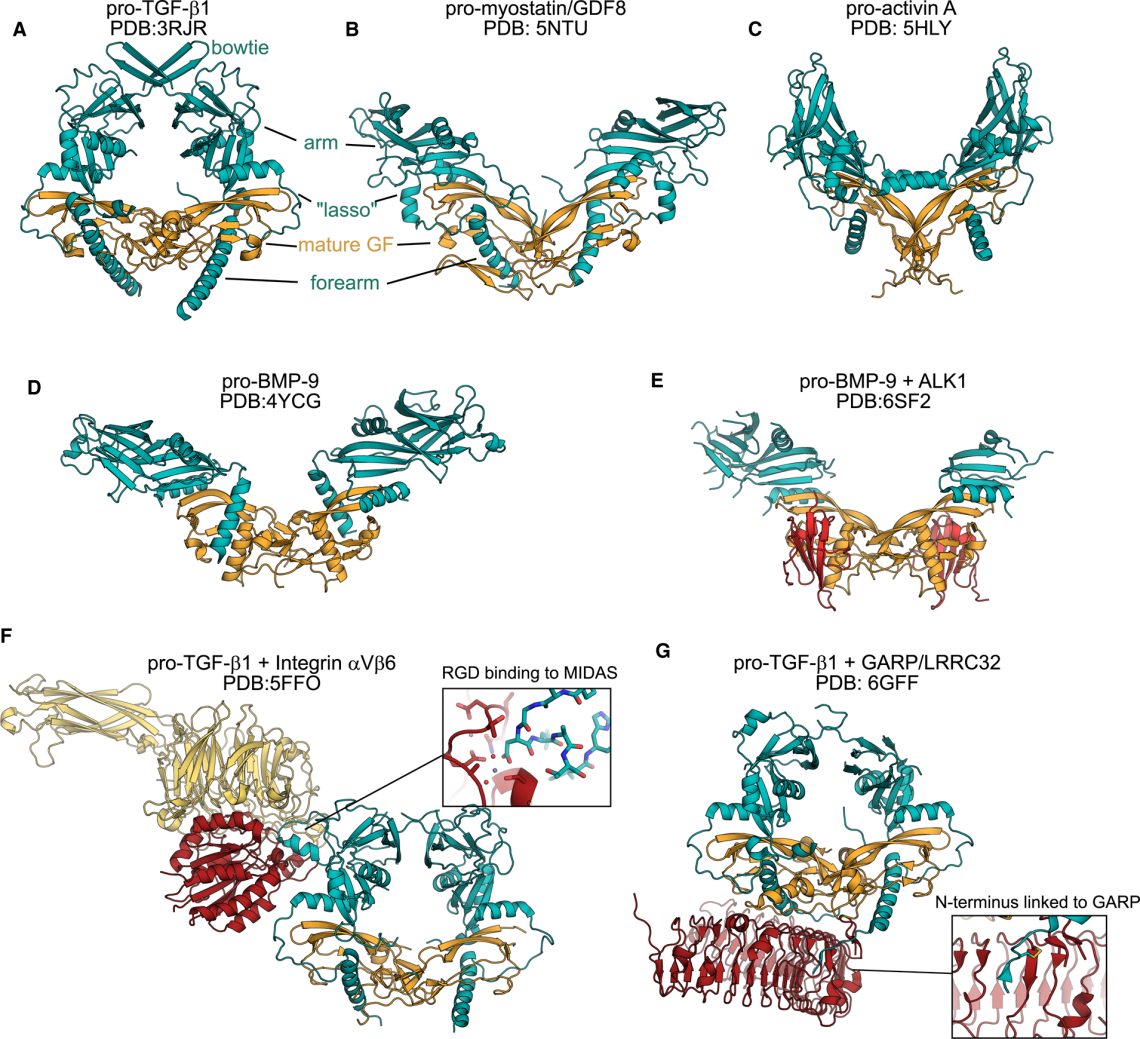
TGF-β family pro-form structures. (**A**) Latent pro-TGF-β (**B**) Pro-myostatin/GDF8 (**C**) Pro-activin A (**D**) Pro-BMP-9 (**E**) Pro-BMP-9 in complex with ALK1 receptor extracellular domain (**F**) Latent pro-TGF-β in complex with integrin αVβ6 extracellular domain with the inset showing the details of the interaction between the integrin metal-ion-dependent adhesion site (MIDAS) domain and the RGD motif on pro-TGF-β1 (**G**). Latent pro-TGF-β in complex with extracellular milieu molecule LRRC32/GARP. The inset showing the details of the interaction between GARP and pro-TGF-β1. The mature domain is in all cases coloured orange and the pro-domains in blue, similar to Figure 1. Other binding proteins are shown in red. Different parts of the pro-domains are labelled for pro-TGF-β1 and pro-myostatin.

Several structures of pro-forms of TGF-β family proteins have been determined experimentally and have helped to understand the role of the pro-domains (Figure 2). While they all contain similar structural elements, the architecture of each complex varies significantly. The N-terminal part of the pro-domain, the so-called fore-arm, forms a long α-helix that interacts with the GF in the so-called wrist epitope where the type I receptors bind. The pro-domain then continues over the type II receptor site of the GF, forming a lasso-like structure, followed by a globular ‘arm’ domain that is bound to the mature GF. The pro-TGF-β1 structure shows a closed complex with arm domains linked by a disulfide in a bowtie-like extension, enclosing the mature ligand and masking all receptor-binding sites (Figure 2A) [8, 9]. Structures of pro-activin A, pro-myostatin and pro-BMP9 have revealed a different architecture, with the globular arm domains of the pro-domains pointing in different orientations, forming open complexes (Figure 2B–D) [10–12]. Structural details of these complexes can be correlated with the level of inhibitory effect of the pro-domains [13].

The prototypical member of this family, TGF-β1, is known to be secreted as a furin-cleaved but still inactive pro-form, the latent-TGF-β1, which can be activated by mechanical force [14]. Myostatin (also called as growth and differentiation factor (GDF)8) is an example of a protein that is secreted in an unprocessed form [15] and remains inhibited by the pro-domain even after cleavage at the furin site [10]. Full activation of myostatin is achieved only after secondary proteolysis by Tolloid-like metalloproteases in the ECM, similar to closely related GDF11 [16, 17]. The pro-form of bone morphogenetic protein 4 (BMP-4) requires also activation by a secondary cleavage of the pro-domain but, in this case, it is at a second furin-like cleavage site that is N-terminal to the primary poly-basic cleavage site [18]. BMP-3A, BMP-3B, GDF-6 and GDF-7 all possess a tribasic cleavage site in addition to the polybasic cleavage site, which generates proteins of different sizes [19–21]. Recently a conserved site for MMP cleavage and GF activation has been found for BMP-7, further widening the mechanisms of activation [22].

Many furin-processed TGF-β growth factors remain complexed with their pro-domains although the pro-domains do not always inhibit their activity. Examples of this include activins, BMP-4, BMP-5, BMP-7 and BMP-9 [11, 23]. Recent structures of pro-BMP-9 and pro-BMP-10 in complex with the extracellular domain of ALK1 receptor demonstrate how pro-domain and ALK1 binding to the mature GF ligands are not mutually exclusive [12]. The same has been observed with pro-BMP-7, where type I receptor and the pro-domain can bind simultaneously, while type II receptors will displace the pro-domain [24].

In addition to influencing GF bioactivity, the pro-domains appear to often play a role in protein localisation. Latent-TGF-βs are the best characterised in this way. These proteins associate with components of the extracellular matrix (ECM) and are disulfide-bonded to latent-TGF-β binding proteins (LTBPs) or Leucine rich repeat containing 32 (LRRC32, aka GARP; Figure 2) [25–28]. These inactive, but furin-cleaved, proteins can activated by diverse mechanism, e.g., by mechanical forces, proteolysis and through interaction with other proteins. Interaction with ECM on one end and with integrin binding to the RGD motif on the other end of the pro-domain, can induce mechanical force that distorts the pro-domain releasing the bioactive mature dimer [14] (Figure 2). Thromospondin-1 can bind both to mature and latent forms of TGF-β and activate the latent form both *in vitro* and *in vivo* [29, 30]. The BMP-4 pro-domain interacts with fibrillin, targeting the complex to the extracellular matrix where the growth factor is stored and stabilised [31]. Pro-domains of other members of the TGF-β superfamily can also interact with components of the ECM at the cell surface. These include the pro-domains of myostatin, which interact with perlecan [23], BMP-4, -5, -7, -10 and GDF-5, which interact with fibrillin-1 and fibrillin-2 [23, 31]. Activin pro-domains are known to bind to heparan sulfates as a potential mechanism for ECM retention [32]. In addition to affecting localisation, interaction with ECM components is very likely to affect activation as well. While pro-activin A complex in solution has the same bioactivity as isolated mature domain, it is likely that pro-domain interactions with heparan sulfate or ECM protein will affect the dissociation of the pro-mature complex [11].

## GDNF family

The glial-derived neurotrophic factor (GNDF) sub-family of TGF-β-like growth factors contains 5 members. While their mature GFs are very similar to classical TGF-β proteins, they have significantly smaller pro-domains, typically just 55–75 residues. These are not predicted to contain a folded domain, and little is known of their structure. GDF15, which has only recently been shown to belong to this group [33], contains a pro-peptide of 154 residues, but it is not known if it forms a stable structure on its own. Pro-GDF15 has been shown to be enriched at the ECM in contrast with the processed mature form, suggesting a localisation role for the GDF15 pro-domain with processing possibly impacting circulating levels of the growth factor [34, 35].

## PDGF/NGF-like growth factors

Platelet-derived growth factors (PDGFs) and nerve growth factor (NGF)-family of GFs contain relatively small pro-peptides, 56–120 residues which are processed by furin-like proprotein convertases. Both NGF and brain-derived neurotrophic factor (BDNF) pro-peptides are known to be necessary for the secretion of the proteins [36]. NGF pro-peptide has been characterised structurally, but it appears not to make well defined interactions with the mature GF even in its uncleaved precursor form, and both pro-domain and mature form are biologically active but display different effect on the target neurons [37]. Pro-NGF has been crystallised in complex with its receptor p75 ectodomain but no interpretable electron density was present for the pro-peptide [38]. In contrast, BDNF pro-peptide has been shown to interact with the mature domain at nanomolar affinity. This interaction is enhanced by pro-peptide variants due to genetic polymorphisms, but structural details of this are missing [39]. Additionally, both NGF and BDNF pro-peptides have been shown to have independent bioactivity. Pro-NGF and pro-BDNF promote apoptosis through p75 and sortilin receptors [40, 41] but specific receptors or mechanisms of signalling for pro-peptides have not been described [37, 42]. Polymorphism in BDNF pro-domain, resulting in Val66Met substitution, is known to affect BDNF processing and secretion and associated with psychiatric disorders [43].

## EGF family

All members of this EGF superfamily, including neuregulins, heparin-binding-EGF (HB-EGF), TGF-α and amphiregulin, are membrane-bound proteins in their precursor form with the mature domain located in the extracellular side. They vary significantly in size, and the pro-domains show little similarity to one another. Except for amphiregulin, EGF-like proteins can also be secreted without the pro-domains [44]. The release of the mature EGF ligands is through a process called ‘ectodomain shedding’ in which typically furin-like and/or ADAM-family proteases cleave the mature ligand from the membrane bound precursor. This process is best characterised for HB-EGF (reviewed in [45]). In addition to release of the extracellular GF, the C-terminal cytoplasmic part of the HB-EGF precursor is also released and translocates to the nucleus [46]. The proteolytic release of EGF is not needed for bioactivity as high molecular weight forms of the protein are also bioactive and can compete with mature EGF for receptor binding [3, 47]. The function of the almost 1000 residue pro-domain of EGF is unknown, apart from containing the transmembrane helix.

## TNF-α family

The TNF-α family members are type II transmembrane proteins with a short cytoplasmic N-terminal pro-domain and the active ligand domain on the extracellular side. Ectodomain shedding releases the trimeric mature TNF-α which can activate its receptor, but the membrane bound form can also signal [2]. The processing of the precursor was identified as a potential mechanism of TNF-α control, and metalloprotease inhibitors [48, 49] helped to identify the processing enzyme TACE (TNF-α converting enzyme, or ADAM17) [50, 51].

## Interleukin-1 family

Of the interleukins, only members of the IL-1 family contain a pro-domain. Of these IL-1β, IL-18 and IL-33, which are all involved in the innate immune system, are particularly interesting as they are strongly up-regulated in a number of human inflammatory diseases [52] and therefore targeting of each of them is being explored as a treatment approach with three therapeutics neutralising IL-1β approved [53]. They all have pro-domains of ∼100 residues, but they lack a signal peptide and hence are not directly secreted from cells. Instead, they are at first localised in the nucleus [54] or move from cytoplasm to nucleus upon stimulus such as cell stress. The release of the mature domain by proteolysis [5] can occur intracellularly or, within vesicles, by calpains or caspases [55]. Different mechanisms of secretion of the mature interleukins have been suggested, including lysosomal vesicles, microvesicles and exosomes [56]. The pro-domains contain a helix-loop-helix motif that is typically found in transcription factors and shown to bind to chromatin where they have been shown to modulate gene expression when overexpressed *in vitro* [57–59]. Nuclear localisation may play a part in the regulation of activity of IL-1 family members, in particular IL-33, as the deletion of the DNA-binding pro-domain results in constitutive activity [60, 61]. The precursors appear to have some bioactivity without processing, but maximal activity is found in the mature protein [62]. The purpose of the pro-domains appears therefore to not only to inhibit activity as such but to regulate release and bio-availability. It is not known what happens to the DNA-binding pro-domain after the mature cytokine has been released.

**Figure 3.**
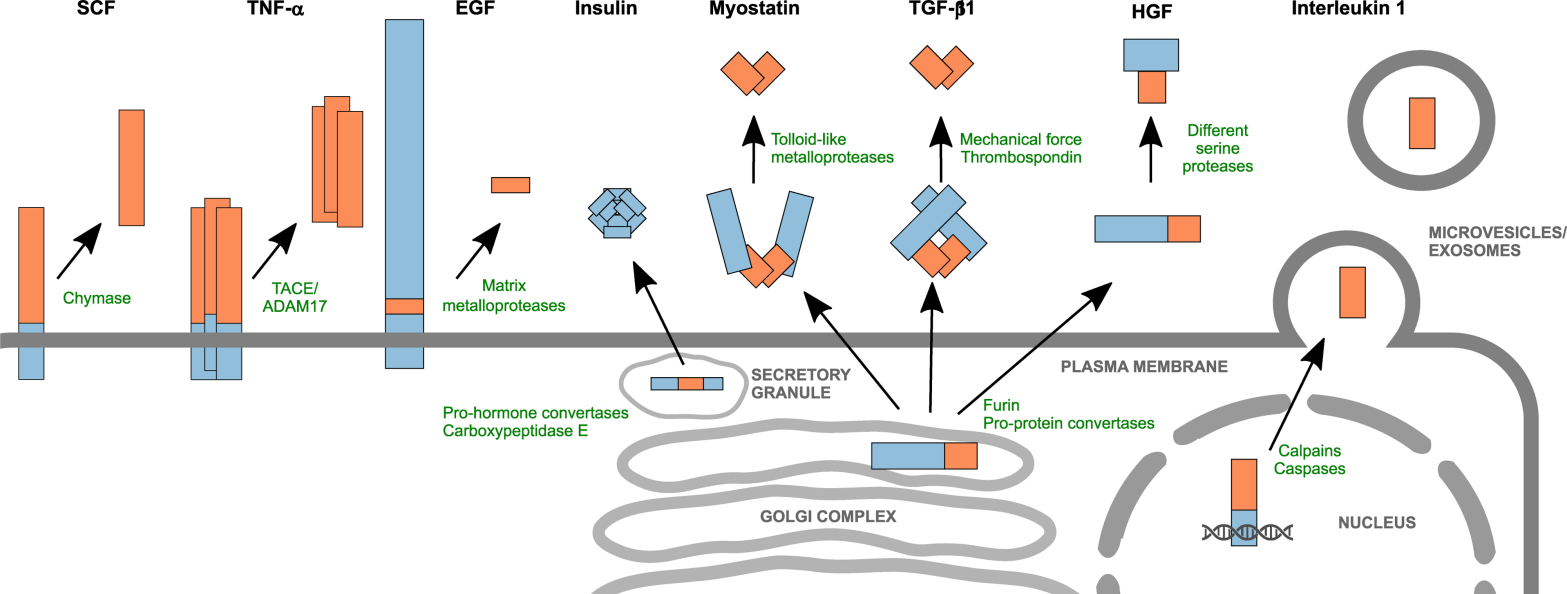
Pro-domain containing growth factors, their activation and localisation. Examples of the diverse cellular localisations and activation processes for representative growth factor and cytokine precursors. Colouring as in Figure 1.

We have summarised the general features of the different families we have discussed in Figure 3, to illustrate the diversity in localisation and processing of these proteins.

## Pro-domain relevance in medicine and drug discovery

A classic example of a pro-domain biomarker use can be seen in insulin. Insulin is produced as a single polypeptide chain, with a pro-domain contained within the middle of the two active chains, A and B, of insulin. This connecting or C-peptide is removed by pro-hormone convertases prior to secretion and released simultaneously and in stoichiometric amounts with active insulin formed out disulfide-linked A and B chains. Insulin C-peptide has been widely used as a proxy for secreted insulin due to its higher serum stability, yet it does appear to also have bioactivity of its own [63].

Some of the GFs have been demonstrated, at least in animal models of disease, to have potential for human therapy [64, 65]. GDNF and the related family member Neurturin for the treatment of Parkinson's disease [66], Neurturin, as a potential key growth hormone in pancreas development, in diabetes, as well as GDF15 for obesity are particularly notable examples. The pharmacological agent is typically the mature GF, but these frequently have unfavourable pharmacological properties, in particular a short serum half-life which in turn significantly limits exposure, that require engineering [67]. Precursors could be considered as alternatives, with different properties and potentially a more controlled release of the mature domain. Pro-domains could also be used, natively or in engineered form, as targeting signals to deliver the GFs to desired part of the body where their bioactivity is needed. Indeed, engineered forms of BMP-2, vascular endothelial growth factor-A (VEGF-A) and platelet-derived growth factor (PDGF) with artificial ECM binding domains, even if not pro-domains in these cases, display improved bioavailability and signalling — leading to enhanced tissue regeneration in animal models [68, 69].

Pro-domains have also inspired a new class of engineered antibody variants, pro-bodies. These are based on masking the binding site of an antibody with a bespoke inhibitory domain that is then tethered to the antibody via a protease-cleavable linker. Only in tissue with the cognate protease present, the linker is then cleaved, leading to release of the masking domain and thereby rendering the pro-body active to bind its cognate antigen. The same principle has also been applied to make variants of cytokines such as IL-2 that are conditionally active only in tissue that expresses the proteases that releases its engineered inhibitory domain [70].

Understanding of pro-domain function can aid in the development of novel therapies that aim to inhibit the mature domain by targeting the proteolytic processing or release of the mature GFs as potential points of intervention. Instead of capturing the free GF after it has been released from the pro-complex and before it binds its receptor, inhibition of ligand activation could be a far more effective strategy. As the GF domains are often highly conserved, antibodies targeting a complex of more diverse pro-domains with mature GFs might also achieve selectivity more easily.

For example, myostatin regulates negatively skeletal muscle growth and its inhibition can treat muscle wasting conditions [71]. Antibodies directly targeting myostatin have been described but were disappointing in clinical studies. Instead, a recent report described antibody-mediated inhibition of proteolytic activation of pro-myostatin. The phage-display selected antibody, SRK-015, binds to the precursor form of myostatin and prevents its proteolysis. SRK-015 binds to the arm region of the pro-domain and protects the protein from proteolytic processing and GF activation [72]. Similarly, antibodies have been generated against TGF-β1 that bind the precursor protein and inhibit its activation, thereby inhibiting TGF-β1 [73].

Pro-domains are also potential inhibitors of GFs themselves. Peptides derived from the first α-helix of myostatin pro-domain are relatively potent inhibitors of myostatin [74, 75], and myostatin pro-domain fused to antibody Fc part can increase muscle mass in *mdx* mice, a mouse model for Duchenne muscular dystrophy [76, 77].

Activin A and B pro-domains, despite their lower intrinsic ability to inhibit bioactivity, have been shown to be potent inhibitors of their respective ligand, especially when driven to dimerise by an Fc domain or by disulfides inspired by latent-TGF-β [78–80].

## Discussion

Growth factor research has historically focussed on mature domains and their pharmacological effects. For TGF-β proteins the importance of the pro-domain for secretion and for ECM localisation has long been described, but for most members of this family any potential biological roles of their pro-domains, if any, are still to be discovered. Peptide hormones are often synthesised as larger precursors which are then processed to yield multiple different hormones with distinct activities, and it would not be unconceivable to think the same is the case with growth factors. Could a pro-domain or -peptide act as a ligand for a yet unidentified receptor? For example, neuropilins recognise peptides with a C-terminal arginine which is present in all pro-domains that have been cleaved by furin-like proteases [81]. Activation of the pro-complexes and ECM-retained pro-growth factors is also still relatively poorly understood. These can act as fast-release depositories of bioactivity, but details of how and when they are activated are still largely unknown.

One of the reasons for the limited research, compared with studies on mature ligands, lies in the lack of tools. We have very few antibodies against pro-domains, reducing the possibilities to study their processing, localisation and fate in tissues and organisms. With antibodies we could also see if pro-domains are useful as serum biomarkers, such as insulin C-peptide. With availability of high-quality antigens for antibody production this sparsity of tools will hopefully be addressed, and the biological roles of pro-domains be elucidated in more detail. The multiplicity of regulation and control of growth factors through their pro-domains and their processing might yet provide a treasure trove of new discoveries on the function and control of growth factor activity as part of tissue homeostasis and disease.

## Perspectives

- Precursors and pro-domains are a greatly understudied aspect of growth factors and cytokines.
- Pro-domains play an important role in the regulation of their mature, active growth factors, but precise mechanisms are still often unresolved.
- Therapeutically pro-domains and precursor forms of growth factors offer possibilities both for modulation of growth factor properties as therapeutic agents and for inhibition of the GFs.

## Open Access Statement

Open access for this article was enabled by the participation of University of Cambridge in an all-inclusive Read & Publish pilot with Portland Press and the Biochemical Society under a transformative agreement with JISC.

## Abbreviations

BDNF: brain-derived neurotrophic factor
BMP-4: bone morphogenetic protein 4
ECM: extracellular matrix
EGF: epidermal growth factor
GDF: growth and differentiation factor
GF: growth factor
HB-EGF: heparin-binding-EGF
HGF: hepatocyte growth factor
NGF: nerve growth factor
TGF-β: transforming growth factor β
TNF-α: tumour necrosis factor α

## References

[1] Bryan, P.N. (2002) Prodomains and protein folding catalysis. Chem. Rev. 102, 4805–4816 10.1021/cr010190b 12475207

[2] Grell, M., Douni, E., Wajant, H., Löhden, M., Clauss, M., Maxeiner, B. et al. (1995) The transmembrane form of tumor necrosis factor is the prime activating ligand of the 80 kDa tumor necrosis factor receptor. Cell 83, 793–802 10.1016/0092-8674(95)90192-2 8521496

[3] Mroczkowski, B. and Reich, M. (1993) Identification of biologically active epidermal growth factor precursor in human fluids and secretions. Endocrinology 132, 417–425 10.1210/endo.132.1.8419140 8419140

[4] Longley, B.J., Tyrrell, L., Ma, Y., Williams, D.A., Halaban, R., Langley, K. et al. (1997) Chymase cleavage of stem cell factor yields a bioactive, soluble product. Proc. Natl Acad. Sci. U.S.A. 94, 9017–9021 10.1073/pnas.94.17.9017 9256427 PMC23007

[5] Carta, S., Lavieri, R. and Rubartelli, A. (2013) Different members of the IL-1 family come out in different ways: DAMPs vs. cytokines? Front. Immunol. 4, 1–9 10.3389/fimmu.2013.00123 23745123 PMC3662868

[6] Kawaguchi, M. and Kataoka, H. (2014) Mechanisms of hepatocyte growth factor activation in cancer tissues. Cancers (Basel 6, 1890–1904 10.3390/cancers6041890 25268161 PMC4276949

[7] Hinck, A.P., Mueller, T.D. and Springer, T.A. (2016) Structural biology and evolution of the TGF-β family. Cold Spring Harb. Perspect. Biol. 8, a022103 10.1101/cshperspect.a022103 27638177 PMC5131774

[8] Zhao, B., Xu, S., Dong, X., Lu, C. and Springer, T.A. (2018) Prodomain-growth factor swapping in the structure of pro-TGF-β1. J. Biol. Chem. 293, 1579–1589 10.1074/jbc.M117.809657 29109152 PMC5798290

[9] Shi, M., Zhu, J., Wang, R., Chen, X., Mi, L., Walz, T. et al. (2011) Latent TGF-β structure and activation. Nature 474, 343–349 10.1038/nature10152 21677751 PMC4717672

[10] Cotton, T.R., Fischer, G., Wang, X., McCoy, J.C., Czepnik, M., Thompson, T.B. et al. (2018) Structure of the human myostatin precursor and determinants of growth factor latency. EMBO J. 37, 367–383 10.15252/embj.201797883 29330193 PMC5793801

[11] Wang, X., Fischer, G. and Hyvönen, M. (2016) Structure and activation of pro-activin A. Nat. Commun. 7, 12052 10.1038/ncomms12052 27373274 PMC4932183

[12] Salmon, R.M., Guo, J., Wood, J.H., Tong, Z., Beech, J.S., Lawera, A. et al. (2020) Molecular basis of ALK1-mediated signalling by BMP9/BMP10 and their prodomain-bound forms. Nat. Commun. 11, 1621 10.1038/s41467-020-15425-3 32238803 PMC7113306

[13] Cotton, T., Fischer, G., Wang, X., Mccoy, J., Czepnik, M. and Thomas, B. (2017) Structure of the human pro-myostatin precursor and determinants of growth factor latency

[14] Buscemi, L., Ramonet, D., Klingberg, F., Formey, A., Smith-Clerc, J., Meister, J.-J. et al. (2011) The single-molecule mechanics of the latent TGF-β1 complex. Curr. Biol. 21, 2046–2054 10.1016/j.cub.2011.11.037 22169532

[15] Pirruccello-Straub, M., Jackson, J., Wawersik, S., Webster, M.T., Salta, L., Long, K. et al. (2018) Blocking extracellular activation of myostatin as a strategy for treating muscle wasting. Sci. Rep. 8, 1–15 10.1038/s41598-018-20524-9 29396542 PMC5797207

[16] Ge, G., Hopkins, D.R., Ho, W. and Greenspan, D.S. (2005) GDF11 forms a bone morphogenetic protein 1-activated latent complex that can modulate nerve growth factor-induced differentiation of PC12 cells. Mol. Cell. Biol. 25, 5846–5858 10.1128/MCB.25.14.5846-5858.2005 15988002 PMC1168807

[17] Le, V.Q., Iacob, R.E., Tian, Y., McConaughy, W., Jackson, J., Su, Y. et al. (2018) Tolloid cleavage activates latent GDF8 by priming the pro-complex for dissociation. EMBO J. 37, 384–397 10.15252/embj.201797931 29343545 PMC5793799

[18] Cui, Y., Hackenmiller, R., Berg, L., Jean, F., Nakayama, T., Thomas, G. et al. (2001) The activity and signaling range of mature BMP-4 is regulated by sequential cleavage at two sites within the prodomain of the precursor. Genes Dev. 15, 2797–2802 10.1101/gad.940001 11691831 PMC312809

[19] Hino, J., Kangawa, K., Matsuo, H., Nohno, T. and Nishimatsu, S. (2004) Bone morphogenetic protein-3 family members and their biological functions. Front. Biosci. 9, 1520–1529 10.2741/1355 14977563

[20] Wolfman, N.M., Hattersley, G., Cox, K., Celeste, A.J., Nelson, R., Yamaji, N. et al. (1997) Ectopic induction of tendon and ligament in rats by growth and differentiation factors 5, 6, and 7, members of the TGF-beta gene family. J. Clin. Invest. 100, 321–330 10.1172/JCI119537 9218508 PMC508194

[21] Williams, L.A., Bhargav, D. and Diwan, A.D. (2008) Unveiling the bmp13 enigma: redundant morphogen or crucial regulator? Int. J. Biol. Sci. 4, 318–329 10.7150/ijbs.4.318 18797508 PMC2536705

[22] Furlan, A.G., Spanou, C.E.S., Godwin, A.R.F., Wohl, A.P., Zimmermann, L.M.A., Imhof, T. et al. (2021) A new MMP-mediated prodomain cleavage mechanism to activate bone morphogenetic proteins from the extracellular matrix. FASEB J. 35, 1–21 10.1096/fj.202001264R PMC12266326 33629769

[23] Sengle, G., Ono, R.N., Sasaki, T. and Sakai, L.Y. (2011) Prodomains of transforming growth factor β (TGFβ) superfamily members specify different functions. J. Biol. Chem. 286, 5087–5099 10.1074/jbc.M110.188615 21135108 PMC3037620

[24] Sengle, G., Ono, R.N., Lyons, K.M., Bächinger, H.P. and Sakai, L.Y. (2008) A new model for growth factor activation: type II receptors compete with the prodomain for BMP-7. J. Mol. Biol. 381, 1025–1039 10.1016/j.jmb.2008.06.074 18621057 PMC2705212

[25] Qin, Y., Garrison, B.S., Ma, W., Wang, R., Jiang, A., Li, J. et al. (2018) A milieu molecule for TGF-β required for microglia function in the nervous system. Cell 174, 156–171.e16 10.1016/j.cell.2018.05.027 29909984 PMC6089614

[26] Liénart, S., Merceron, R., Vanderaa, C., Lambert, F., Colau, D., Stockis, J. et al. (2018) Structural basis of latent TGF-β1 presentation and activation by GARP on human regulatory T cells. Science 362, 952–956 10.1126/science.aau2909 30361387

[27] Campbell, M.G., Cormier, A., Ito, S., Seed, R.I., Bondesson, A.J., Lou, J. et al. (2020) Cryo-EM reveals integrin-mediated TGF-β activation without release from latent TGF-β. Cell 180, 490–501.e16 10.1016/j.cell.2019.12.030 31955848 PMC7238552

[28] Rifkin, D.B. (2005) Latent transforming growth factor-beta (TGF-beta) binding proteins: orchestrators of TGF-beta availability. J. Biol. Chem. 280, 7409–7412 10.1074/jbc.R400029200 15611103

[29] Schultz-Cherry, S., Ribeiro, S., Gentry, L. and Murphy-Ullrich, J.E. (1994) Thrombospondin binds and activates the small and large forms of latent transforming growth factor-beta in a chemically defined system. J. Biol. Chem. 269, 26775–26782 10.1016/S0021-9258(18)47086-X 7929413

[30] Crawford, S.E., Stellmach, V., Murphy-Ullrich, J.E., Ribeiro, S.M., Lawler, J., Hynes, R.O. et al. (1998) Thrombospondin-1 is a major activator of TGF-β1 *in vivo*. Cell 93, 1159–1170 10.1016/S0092-8674(00)81460-9 9657149

[31] Sengle, G., Charbonneau, N.L., Ono, R.N., Sasaki, T., Alvarez, J., Keene, D.R. et al. (2008) Targeting of bone morphogenetic protein growth factor complexes to fibrillin. J. Biol. Chem. 283, 13874–13888 10.1074/jbc.M707820200 18339631 PMC2376219

[32] Li, S., Shimono, C., Norioka, N., Nakano, I., Okubo, T., Yagi, Y. et al. (2010) Activin A binds to perlecan through its pro-region that has heparin/heparan sulfate binding activity. J. Biol. Chem. 285, 36645–36655 10.1074/jbc.M110.177865 20843788 PMC2978593

[33] Mullican, S.E., Lin-Schmidt, X., Chin, C.-N., Chavez, J.A., Furman, J.L., Armstrong, A.A. et al. (2017) GFRAL is the receptor for GDF15 and the ligand promotes weight loss in mice and nonhuman primates. Nat. Med. 23, 1150–1157 10.1038/nm.4392 28846097

[34] Bauskin, A.R., Jiang, L., Luo, X.W., Wu, L., Brown, D.A. and Breit, S.N. (2010) The TGF-β superfamily cytokine MIC-1/GDF15: Secretory mechanisms facilitate creation of latent stromal stores. J. Interf. Cytokine Res. 30, 389–397 10.1089/jir.2009.0052 20187768

[35] Bauskin, A.R., Brown, D.A., Junankar, S., Rasiah, K.K., Eggleton, S., Hunter, M. et al. (2005) The propeptide mediates formation of stromal stores of PROMIC-1: role in determining prostate cancer outcome. Cancer Res. 65, 2330–2336 10.1158/0008-5472.CAN-04-3827 15781647

[36] Suter, U., Heymach J, V. and Shooter, E.M. (1991) Two conserved domains in the NGF propeptide are necessary and sufficient for the biosynthesis of correctly processed and biologically active NGF. EMBO J. 10, 2395–2400 10.1002/j.1460-2075.1991.tb07778.x 1868828 PMC452934

[37] Yan, R., Yalinca, H., Paoletti, F., Gobbo, F., Marchetti, L., Kuzmanic, A. et al. (2019) The structure of the Pro-domain of mouse proNGF in contact with the NGF domain. Structure 27, 78–89.e3 10.1016/j.str.2018.09.013 30393051

[38] Feng, D., Kim, T., Ozkan, E., Light, M., Torkin, R., Teng, K.K. et al. (2010) Molecular and structural insight into proNGF engagement of p75NTR and sortilin. J. Mol. Biol. 396, 967–984 10.1016/j.jmb.2009.12.030 20036257 PMC2847487

[39] Uegaki, K., Kumanogoh, H., Mizui, T., Hirokawa, T., Ishikawa, Y. and Kojima, M. (2017) BDNF binds its pro-peptide with high affinity and the common Val66Met polymorphism attenuates the interaction. Int. J. Mol. Sci. 18, 1042 10.3390/ijms18051042 PMC5454954 28498321

[40] Teng, H.K., Teng, K.K., Lee, R., Wright, S., Tevar, S., Almeida, R.D. et al. (2005) ProBDNF induces neuronal apoptosis via activation of a receptor complex of p75NTR and sortilin. J. Neurosci. 25, 5455–5463 10.1523/JNEUROSCI.5123-04.2005 15930396 PMC6724992

[41] Nykjaer, A., Lee, R., Teng, K.K., Jansen, P., Madsen, P., Nielsen, M.S. et al. (2004) Sortilin is essential for proNGF-induced neuronal cell death. Nature 427, 843–848 10.1038/nature02319 14985763

[42] Mizui, T., Ishikawa, Y., Kumanogoh, H., Lume, M., Matsumoto, T., Hara, T. et al. (2015) BDNF pro-peptide actions facilitate hippocampal LTD and are altered by the common BDNF polymorphism Val66Met. Proc. Natl Acad. Sci. U.S.A. 112, E3067–E3074 10.1073/pnas.1422336112 26015580 PMC4466729

[43] Notaras, M., Hill, R. and Van Den Buuse, M. (2015) The BDNF gene Val66Met polymorphism as a modifier of psychiatric disorder susceptibility: Progress and controversy. Mol. Psychiatry 20, 916–930 10.1038/mp.2015.27 25824305

[44] Thorne, B.A. and Plowman, G.D. (1994) The heparin-binding domain of amphiregulin necessitates the precursor pro-region for growth factor secretion. Mol. Cell. Biol. 14, 1635–1646 10.1128/mcb.14.3.1635-1646.1994 8114701 PMC358522

[45] Taylor, S.R., Markesbery, M.G. and Harding, P.A. (2014) Heparin-binding epidermal growth factor-like growth factor (HB-EGF) and proteolytic processing by a disintegrin and metalloproteinases (ADAM): A regulator of several pathways. Semin. Cell Dev. Biol. 28, 22–30 10.1016/j.semcdb.2014.03.004 24680771

[46] Nanba, D., Mammoto, A., Hashimoto, K. and Higashiyama, S. (2003) Proteolytic release of the carboxy-terminal fragment of proHB-EGF causes nuclear export of PLZF. J. Cell Biol. 163, 489–502 10.1083/jcb.200303017 14597771 PMC2173632

[47] Mroczkowski, B., Reich, M., Chen, K., Bell, G.I. and Cohen, S. (1989) Recombinant human epidermal growth factor precursor is a glycosylated membrane protein with biological activity. Mol. Cell. Biol. 9, 2771–2778 10.1128/mcb.9.7.2771-2778.1989 2789334 PMC362742

[48] McGeehan, G.M., Becherer, J.D., Bast, R.C., Boyer, C.M., Champion, B., Connolly, K.M. et al. (1994) Regulation of tumour necrosis factor-α processing by a metalloproteinase inhibitor. Nature 370, 558–561 10.1038/370558a0 8052311

[49] Mohler, K.M., Sleath, P.R., Fitzner, J.N., Cerretti, D.P., Alderson, M., Kerwar, S.S. et al. (1994) Protection against a lethal dose of endotoxin by an inhibitor of tumour necrosis factor processing. Nature 370, 218–220 10.1038/370218a0 8028669

[50] Black, R.A., Rauch, C.T., Kozlosky, C.J., Peschon, J.J., Slack, J.L., Wolfson, M.F. et al. (1997) A metalloproteinase disintegrin that releases tumour-necrosis factor-α from cells. Nature 385, 729–733 10.1038/385729a0 9034190

[51] Moss, M.L., Jin, S.L.C., Milla, M.E., Burkhart, W., Carter, H.L., Chen, W.J. et al. (1997) Cloning of a disintegrin metalloproteinase that processes precursor tumour-necrosis factor-α. Nature 385, 733–736 10.1038/385733a0 9034191

[52] Dinarello, C.A. (2018) Overview of the IL-1 family in innate inflammation and acquired immunity. Immunol. Rev. 281, 8–27 10.1111/imr.12621 29247995 PMC5756628

[53] Junge, G., Mason, J. and Feist, E. (2017) Adult onset still's disease—The evidence that anti-interleukin-1 treatment is effective and well-tolerated (a comprehensive literature review). Semin. Arthritis Rheum. 47, 295–302 10.1016/j.semarthrit.2017.06.006 28757235

[54] Maier, J.A., Statuto, M. and Ragnotti, G. (1994) Endogenous interleukin 1 alpha must be transported to the nucleus to exert its activity in human endothelial cells. Mol. Cell. Biol. 14, 1845–1851 10.1128/mcb.14.3.1845-1851.1994 8114717 PMC358542

[55] Afonina, I.S., Müller, C., Martin, S.J. and Beyaert, R. (2015) Proteolytic processing of interleukin-1 family cytokines: variations on a common theme. Immunity. 42, 991–1004 10.1016/j.immuni.2015.06.003 26084020

[56] Lopez-Castejon, G. and Brough, D. (2011) Understanding the mechanism of IL-1β secretion. Cytokine Growth Factor Rev. 22, 189–195 10.1016/j.cytogfr.2011.10.001 22019906 PMC3714593

[57] Werman, A., Werman-Venkert, R., White, R., Lee, J.K., Werman, B., Krelin, Y. et al. (2004) The precursor form of IL-1α is an intracrine proinflammatory activator of transcription. Proc. Natl Acad. Sci. U.S.A. 101, 2434–2439 10.1073/pnas.0308705101 14983027 PMC356968

[58] Stevenson, F.T., Turck, J., Locksley, R.M. and Lovett, D.H. (1997) The N-terminal propiece of interleukin 1α is a transforming nuclear oncoprotein. Proc. Natl Acad. Sci. U.S.A. 94, 508–513 10.1073/pnas.94.2.508 9012814 PMC19543

[59] Pollock, A.S., Turck, J. and Lovett, D.H. (2003) The prodomain of interleukin 1α interacts with elements of the RNA processing apparatus and induces apoptosis in malignant cells. FASEB J. 17, 203–213 10.1096/fj.02-0602com 12554699

[60] Bessa, J., Meyer, C.A., de Vera Mudry, M.C., Schlicht, S., Smith, S.H., Iglesias, A. et al. (2014) Altered subcellular localization of IL-33 leads to non-resolving lethal inflammation. J. Autoimmun. 55, 33–41 10.1016/j.jaut.2014.02.012 24786898

[61] Hong, J., Bae, S., Jhun, H., Lee, S., Choi, J., Kang, T. et al. (2011) Identification of constitutively active interleukin 33 (IL-33) splice variant. J. Biol. Chem. 286, 20078–20086 10.1074/jbc.M111.219089 21454686 PMC3103380

[62] Mosley, B., Dower, S.K., Gillis, S. and Cosman, D. (1987) Determination of the minimum polypeptide lengths of the functionally active sites of human interleukins 1 alpha and 1 beta. Proc. Natl Acad. Sci. U.S.A. 84, 4572–4576 10.1073/pnas.84.13.4572 2955410 PMC305132

[63] Hills, C.E. and Brunskill, N.J. (2009) Cellular and physiological effects of C-peptide. Clin. Sci. 116, 565–574 10.1042/CS20080441 19243312

[64] Trevaskis, J.L., Sacramento, C.B., Jouihan, H., Ali, S., Le Lay, J., Oldham, S. et al. (2017) Neurturin and a GLP-1 analogue act synergistically to alleviate diabetes in zucker diabetic fatty rats. Diabetes 66, 2007–2018 10.2337/db16-0916 28408435

[65] Hale, C. and Véniant, M.M. (2021) Growth differentiation factor 15 as a potential therapeutic for treating obesity. Mol. Metab. 46, 101117 10.1016/j.molmet.2020.101117 33220493 PMC8085570

[66] Barker, R.A., Björklund, A., Gash, D.M., Whone, A., Van Laar, A., Kordower, J.H. et al. (2020) GDNF and Parkinson's disease: where next? A summary from a recent workshop. J. Parkinsons Dis. 10, 875–891 10.3233/JPD-202004 32508331 PMC7458523

[67] Runeberg-Roos, P., Piccinini, E., Penttinen, A.-M., Mätlik, K., Heikkinen, H., Kuure, S. et al. (2016) Developing therapeutically more efficient neurturin variants for treatment of Parkinson's disease. Neurobiol. Dis. 96, 335–345 10.1016/j.nbd.2016.07.008 27425888

[68] Mochizuki, M., Güç, E., Park, A.J., Julier, Z., Briquez, P.S., Kuhn, G.A. et al. (2020) Growth factors with enhanced syndecan binding generate tonic signalling and promote tissue healing. Nat. Biomed. Eng. 4, 463–475 10.1038/s41551-019-0469-1 31685999

[69] Martino, M.M., Briquez, P.S., Guc, E., Tortelli, F., Kilarski, W.W., Metzger, S. et al. (2014) Growth factors engineered for super-affinity to the extracellular matrix enhance tissue healing. Science 343, 885–888 10.1126/science.1247663 24558160

[70] Kavanaugh, W.M. (2020) Antibody prodrugs for cancer. Expert Opin. Biol. Ther. 20, 163–171 10.1080/14712598.2020.1699053 31779489

[71] Smith, R.C. and Lin, B.K. (2013) Myostatin inhibitors as therapies for muscle wasting associated with cancer and other disorders. Curr. Opin. Support. Palliat. Care 7, 352–360 10.1097/SPC.0000000000000013 24157714 PMC3819341

[72] Dagbay, K.B., Treece, E., Streich, F.C., Jackson, J.W., Faucette, R.R., Nikiforov, A. et al. (2020) Structural basis of specific inhibition of extracellular activation of pro- or latent myostatin by the monoclonal antibody SRK-015. J. Biol. Chem. 295, 5404–5418 10.1074/jbc.RA119.012293 32075906 PMC7170532

[73] Martin, C.J., Datta, A., Littlefield, C., Kalra, A., Chapron, C., Wawersik, S. et al. (2020) Selective inhibition of TGFβ1 activation overcomes primary resistance to checkpoint blockade therapy by altering tumor immune landscape. Sci. Transl. Med. 12, eaay8456 10.1126/scitranslmed.aay8456 32213632

[74] Takayama, K., Rentier, C., Asari, T., Nakamura, A., Saga, Y., Shimada, T. et al. (2017) Development of potent myostatin inhibitory peptides through hydrophobic residue-directed structural modification. ACS Med. Chem. Lett. 8, 751–756 10.1021/acsmedchemlett.7b00168 28740611 PMC5512132

[75] Asari, T., Takayama, K., Nakamura, A., Shimada, T., Taguchi, A. and Hayashi, Y. (2017) Structural basis for the effective myostatin inhibition of the mouse myostatin prodomain-derived minimum peptide. ACS Med. Chem. Lett. 8, 113–117 10.1021/acsmedchemlett.6b00420 28105285 PMC5238466

[76] Bogdanovich, S., Perkins, K.J., Krag, T.O.B., Whittemore, L.-A. and Khurana, T.S. (2005) Myostatin propeptide-mediated amelioration of dystrophic pathophysiology. FASEB J. 19, 543–549 10.1096/fj.04-2796com 15791004

[77] Qiao, C., Li, J., Jiang, J., Zhu, X., Wang, B., Li, J. et al. (2008) Myostatin propeptide gene delivery by adeno-associated virus serotype 8 vectors enhances muscle growth and ameliorates dystrophic phenotypes in mdx mice. Hum. Gene Ther. 19, 241–254 10.1089/hum.2007.159 18288893

[78] Walton, K.L., Chen, J.L., Arnold, Q., Kelly, E., La, M., Lu, L. et al. (2019) Activin A-induced cachectic wasting is attenuated by systemic delivery of its cognate propeptide in male mice. Endocrinology 160, 2417–2426 10.1210/en.2019-00257 31322699

[79] Chen, J.L., Walton, K.L., Hagg, A., Colgan, T.D., Johnson, K., Qian, H. et al. (2017) Specific targeting of TGF-β family ligands demonstrates distinct roles in the regulation of muscle mass in health and disease. Proc. Natl Acad. Sci. U.S.A. 114, E5266–E5275 10.1073/pnas.1620013114 28607086 PMC5495232

[80] Chen, J.L., Walton, K.L., Al-Musawi, S.L., Kelly, E.K., Qian, H., La, M. et al. (2015) Development of novel activin-targeted therapeutics. Mol. Ther. 23, 434–444 10.1038/mt.2014.221 25399825 PMC4351455

[81] Parker, M.W., Xu, P., Li, X. and Vander Kooi, C.W. (2012) Structural basis for selective vascular endothelial growth factor-A (VEGF-A) binding to neuropilin-1. J. Biol. Chem. 287, 11082–11089 10.1074/jbc.M111.331140 22318724 PMC3322888

